# Diabetes mellitus and the neutrophil to lymphocyte ratio predict overall survival in non-viral hepatocellular carcinoma treated with transarterial chemoembolization

**DOI:** 10.3892/ol.2014.1896

**Published:** 2014-02-20

**Authors:** JIANGGUO ZHANG, FENGYUN GONG, LING LI, MANZHI ZHAO, JIANXIN SONG

**Affiliations:** 1 Department of Infectious Diseases, Tongji Hospital, Huazhong University of Science and Technology, Wuhan, Hubei 430030, P.R. China; 2 Department of Infectious Diseases, The Fourth Hospital of Wuhan, Wuhan, Hubei 430030, P.R. China

**Keywords:** non-viral, hepatocellular carcinoma, diabetes mellitus, neutrophil to lymphocyte ratio, chemoembolization, overall survival

## Abstract

Diabetes mellitus (DM) and systemic inflammation are closely associated with the development of hepatocellular carcinoma (HCC). However, the prognostic significance of DM on HCC remains controversial. The main purpose of the present study was to evaluate the effects of DM and the systemic inflammation-based neutrophil to lymphocyte ratio (NLR) on the overall survival (OS) rate of non-viral HCC patients treated with transarterial chemoembolization (TACE). A retrospective analysis of 138 patients with HCC, who were diagnosed between 2002 and 2012 with non-viral causes and who later underwent TACE, was performed. Among these patients, 34 (24.6%) had pre-existing DM and 46 (33.3%) exhibited an elevated baseline NLR (≥5). The multivariate analysis showed that DM, the NLR and a portal vein tumor thrombus (PVTT) were independent predictors for a poor OS rate (P<0.05). The patients with DM and an elevated NLR exhibited a poorer OS rate when compared with patients without these factors. In addition, there was a significant stepwise improvement in the OS rate of patients with DM and an elevated NLR, and in patients with only one of these factors compared with patients without either (P<0.01). Finally, DM was significantly correlated with PVTT and elevated γ-glutamyl transpeptidase levels, while the NLR was independently associated with PVTT and tumor multiplicity (P<0.05). The present study revealed that DM, baseline NLR and PVTT are independent indicators of the OS rate in non-viral HCC patients treated with TACE. DM and NLR may affect the OS rate by promoting the malignant progression of HCC. The combination of DM and NLR appears to be a stronger predictor for OS than DM or NLR alone.

## Introduction

Hepatocellular carcinoma (HCC), the sixth most common cancer on a global scale, is the third major cause of cancer-related mortalities and causes ~598,000 fatalities annually (1). In recent years, ~81.5% of HCCs have been correlated with chronic viral infection [hepatitis B virus (HBV) and hepatitis C virus (HCV) infection], whereas 18.5% have been associated with non-viral causes [HBV surface antigen (HBsAg)-negative and HCV antibody (Ab)-negative] in mainland China (2). For areas in which HBV infection is prevalent, including China, the HBV vaccine has dramatically decreased the incidence of HBV-related HCC (3). Recent developments in the management of patients infected with HBV and/or HCV by specific antiviral therapy, including interferon and nucleotide analogues, has also led to a decrease of viral infection-related HCC development and improved its prognosis (4,5). However, with the decrease of HBV and/or HCV-related HCC, the incidence of non-viral HCC is gradually increasing (6).

It has been estimated that 60–70% of patients with HCC present with an intermediate to advanced stage of disease at diagnosis, and the majority of the tumors are unresectable, particularly the non-viral HCC patients who have not undergone a surveillance program for viral-related HCC (7,8). Transarterial chemoembolization (TACE) is the first-line of treatment for unresectable HCC, and randomized controlled trials have confirmed its benefits in improving the median survival rate (9).

Recent studies have shown that components of metabolic syndrome, including diabetes mellitus (DM), obesity and hyperlipidemia, are significant risk factors for non-viral HCC (10), and the presence of DM at baseline is highly associated with the development of non-viral HCC (11). Insulin resistance, which plays a crucial role in the pathogenesis of DM, is closely associated with the carcinogenesis of HCC through high energy intake, increased cell proliferation and the suppression of apoptosis (12). In addition, systemic inflammation, which can induce insulin resistance through reducing insulin sensitivity, is also involved in various stages of HCC tumorigenesis (13–15). Further studies have demonstrated that pre-existing DM can worsen the outcome of patients with HCC undergoing surgical and non-surgical therapy (16–18), and that systemic inflammation markers, including the neutrophil to lymphocyte ratio (NLR), can also predict the survival of HCC patients (19). However, little information has been focused on the effect of pre-existing DM and baseline NLR on the overall survival (OS) rate of non-viral HCC. Therefore, it would be important to elucidate the association between pre-existing DM, baseline NLR and OS in non-viral HCC. In addition, the significance of pre-existing DM and baseline NLR in non-viral HCC survival has not yet been explicitly explored.

The present study examined the clinical value of pre-existing DM and the NLR, which were measured when patients with non-viral HCC undergoing TACE were enrolled in a large number. The present findings also evaluated the combined prognostic value of pre-existing DM and NLR in predicting the OS rate of non-viral HCC patients. Additionally, the correlation between pre-existing DM, baseline NLR and other clinical pathological factors of non-viral HCC was identified.

## Patients and methods

### Patient selection

A total of 138 patients with non-viral HCC that was newly diagnosed between March 2002 and August 2012 at Tongji Hospital, Huazhong University of Science and Technology (Wuhan, Hubei, China) were prospectively collected and retrospectively analyzed. HCC was diagnosed on the basis of a liver biopsy or clinical criteria, including dynamic computed tomography (CT) images and magnetic resonance imaging (MRI), with or without elevated serum α-fetoprotein (AFP) levels (>200 ng/ml) (20). The patients with non-viral HCC were defined as those who had serum that was negative for HBsAg and HCV Ab. All the non-viral HCC patients received TACE treatment. The present study was approved by the Ethics Committees of Huazhong University of Science and Technology in accordance with the ethical guidelines of the 1975 Declaration of Helsinki. Consent was obtained from either the patient or the patient’s family.

### TACE procedure

TACE treatment was a type of intra-arterial chemotherapy using three combinations of 40 mg cisplatin, 6 mg mitomycin C and 1,000 mg 5-fluorouracil, while the embolization agents used were gelatin sponges and lipiodol (5–20 ml, according to the tumor size).

### Clinicopathological variables and follow-up

Demographic data, including age and gender, were collected at the time of diagnosis. Hematological data, including neutrophil, lymphocyte and platelet counts, and biochemical data, including aspartate aminotransferase (AST), alanine aminotransferase (ALT), γ-glutamyl transpeptidase (GGT), alkaline phosphatase (ALP), albumin, total bilirubin (TBIL), AFP, total cholesterol and blood glucose levels and the international normalized ratio (INR), were measured using an Abbott Aeroset AutoAnalyzer (Abbott Diagnostics, Abbott Park, IL, USA) according to standard techniques for each patient at the time of HCC diagnosis. Anti-HCV Ab and HBsAg in sera were assayed using the EIA Cobas Core Test (Hoffmann-La Roche, Ltd., Basel, Switzerland).

The diagnosis of pre-existing DM was based on the presence of a fasting plasma glucose level of ≥7.0 mmol/l on at least two occasions, a 2-h plasma glucose of ≥11.1 mmol/l in a 75-g oral glucose tolerance test or the requirement for oral hypoglycemic agents and/or insulin to control glucose levels. The NLR was calculated by dividing the neutrophil count by the lymphocyte count, and NLR measurements were obtained without obvious infection. An elevated NLR was defined as ≥5, in agreement with a previous study (19). The presence of a portal vein tumor thrombus (PVTT) was detected by means of ultrasound, a contrast-enhanced CT scan or MRI. The maximum tumor diameter and number of tumors were measured by means of ultrasound, dynamic CT scan or MRI. Hypertension was defined as a systolic blood pressure of >140 mmHg or a diastolic blood pressure of >90 mmHg on two occasions in the medical record of the patient. The Child-Turcotte-Pugh classification, which is based on the serum levels of bilirubin and albumin, prothrombin time prolongation and the severity of encephalopathy and ascites, was assessed. An elevated serum GGT was defined as >50 IU/l (normal range, 11–50 IU/l) and an elevated serum ALP was >150 U/l (normal range, 40–150 U/l). For surveillance of the OS rate of the patients with HCC following TACE treatment, the time of the initial TACE treatment was defined as day zero and patients were evaluated every one to three months.

### Statistical analysis

The differences between groups were analyzed using Pearson’s χ^2^ test for the categorical variables, and a one-way analysis of variance was used for the continuous variables. Cox’s proportional hazard model was applied to explore the independent prognostic value of each variable. A survival analysis of the various clinical factors was carried out using Kaplan-Meier statistics and compared by log-rank test. The correlation between two variables was examined by Spearman’s correlation analysis. All statistical analyses were conducted using SPSS, version 13.0 (SPSS, Inc., Chicago, IL, USA). P<0.05 was considered to indicate a statistically significant difference.

## Results

### Baseline characteristics

Between 2002 and 2012, a total of 138 patients with non-viral HCC, who underwent initial treatment of TACE, were recruited. All clinical variables were obtained from patients at baseline at the time of recruitment into the study, and baseline characteristics are shown in Table I. The mean age was 56.8±12.5 years and the majority of patients were male (n=99, 71.7%). At diagnosis, the number of patients with pre-existing DM and elevated NLR was 34 (24.6%) and 46 (33.3%), respectively. In total, 105 (76.1%) patients were classified as Child-Pugh class A, while 33 (23.9%) patients belonged to Child-Pugh class B. The mean maximum tumor diameter was 8.7±4.5 cm and the number of patients with a solitary tumor was 63 (45.7%). The presence of a PVTT was recorded in 67 (48.6%) patients. The number of patients who received one session of TACE was 115 (83.3%), whereas the number who received >1 session was 23 (16.7%). The mean levels of serum ALT, AST, ALB, TBIL, ALP, GGT, AFP, PLT, INR and NLR were 51.7±67.1 U/l, 70.6±85.2 U/l, 38.9±5.3 g/l, 17.9±24.8 μmol/l, 200.4±170.3 U/l, 201.0±230.0 U/l, 7697.3±21041.3 ng/ml, (189.9±96.1)x10^9^/l, 1.06±0.11 and 4.55±2.71, respectively.

### Prognostic factors affecting the survival of patients with non-viral HCC

To identify the variables affecting the survival rate of patients with non-viral HCC, the factors that exhibited a potential impact on the prognosis of non-viral HCC were examined (Table II). Of those factors, elevated GGT [P=0.04; hazard ratio (HR), 1.519; 95% confidence interval (CI), 1.019–2.265], tumor size (P=0.014; HR, 1.723; 95% CI, 1.114–2.665), tumor multiplicity (P=0.03; HR, 1.483; 95% CI, 1.038–2.119), presence of a PVTT (P<0.001; HR, 4.336; 95% CI, 2.955–6.363), pre-existing DM (P=0.003; HR, 1.868; 95% CI, 1.239–2.816) and an elevated baseline NLR (P<0.001; HR 2.136; 95% CI, 1.466–3.114) were significantly associated with poorer survival in the univariate analysis. The multivariate analysis showed that the presence of a PVTT (P<0.001; HR, 4.235; 95% CI, 2.787–6.436), pre-existing DM (P=0.006; HR, 1.843; 95% CI, 1.190–2.854) and an elevated NLR (P<0.001; HR, 2.126; 95% CI, 1.429–3.165) were identified as independent poor prognostic factors for patients with non-viral HCC who underwent TACE. When the combination of pre-existing DM and elevated NLR was analyzed as one factor, the combination of DM and NLR (P<0.001; HR, 2.235; 95% CI, 1.488–3.357) together with the presence of a PVTT (P<0.001; HR, 4.466; 95% CI, 2.924–6.822) were identified as independent factors for poor survival compared with pre-existing DM or an elevated NLR alone.

The prognostic value of the other metabolic components, including the presence of hypercholesterolemia and hypertension, was also examined in Cox’s regression model, and no statistically significant difference between the presence of hypercholesterolemia and hypertension and the absence of these factors was observed in the univariate analysis (P>0.05).

### Correlation between DM, NLR and clinical factors of non-viral HCC patients

The presence of a PVTT in the patients with DM was significantly increased compared with the patients without DM (67.6 vs. 42.3%). In contrast to patients with an elevated NLR, patients with a normal NLR exhibited a significantly decreased presence of a PVTT (42.4 vs. 60.9%) and more single liver tumors (52.2 vs. 32.6%), respectively.

The association between PVTT, elevated GGT and pre-existing DM was further examined (Table III). The result demonstrated that pre-existing DM was significantly correlated with the presence of a PVTT (Spearman’s ρ, 0.218; P=0.01) and elevated GGT (Spearman’s ρ, 0.186; P=0.029). The correlation of the presence of a PVTT and the number of tumors with an elevated NLR was also examined (Table III). The result identified that an elevated NLR significantly correlated with the presence of a PVTT (Spearman’s ρ, 0.174; P=0.041) and multiple tumors (Spearman’s ρ, 0.185; P=0.03).

### OS according to DM status, NLR level and PVTT presence in non-viral HCC patients

**Figure 1:**
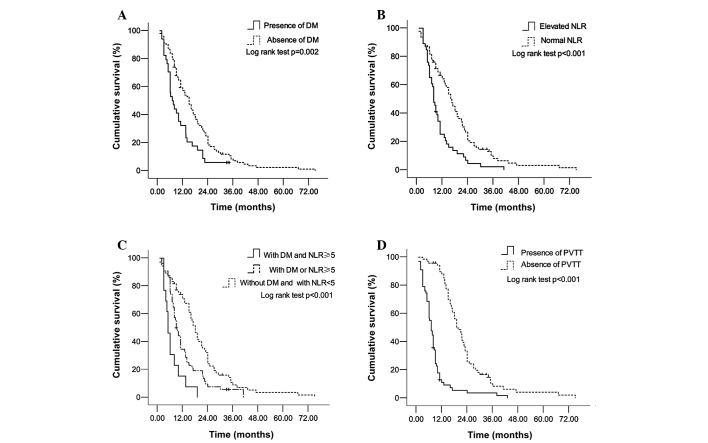
Kaplan-Meier survival analysis of non-viral HCC patients stratified by (A) DM, (B) NLR, (C) a combination of DM and NLR and (D) PVTT. PVTT, portal vein tumor thrombus; DM, diabetes mellitus; NLR, neutrophil to lymphocyte ratio; HCC, hepatocellular carcinoma.

The median follow-up period was 12 months (range, 2–76 months) for the non-viral HCC patients. Since pre-existing DM, elevated baseline NLR and the presence of a PVTT independently predicted the OS rate of non-viral HCC in the multivariate analysis, the differences in survival rate between the presence and absence of DM, elevated and normal NLR and the presence and absence of a PVTT among the whole group were evaluated. As described in Fig. 1A, the OS rate among patients with pre-existing DM (1-year cumulative survival rate, 32.4%; 3-year cumulative survival rate, 0%; and 5-year cumulative survival rate, 0%) was significantly lower than among patients without DM (1-year survival rate, 57.3%; 3-year survival rate, 7.1%; and 5-year survival rate, 1.2%) (log-rank test, P=0.002). Similarly, the survival rate in the elevated NLR group (1-year survival rate, 25.2%; 3-year survival rate, 2.3%; and 5-year survival rate, 0%) was significantly lower when compared with the normal NLR group (1-year survival rate, 64.3%; 3-year survival rate, 8.2%; and 5-year survival rate, 1.6%) (log-rank test, P<0.001; Fig. 1B). A specific Kaplan-Meier analysis was performed (Fig. 1C) to detect the effect of a combined use of pre-existing DM and NLR on patient survival. There was a significant stepwise improvement in the OS rate of patients with pre-existing DM and elevated NLR, with pre-existing DM or elevated NLR and with an absence of DM and normal NLR (log-rank test, P<0.001). This revealed the advantage of the combined use of pre-existing DM and elevated NLR in non-viral HCC patients. Finally, the survival time of patients with the presence of a PVTT was examined (Fig. 1D), and it was shown that these patients exhibited a significantly poorer survival rate (1-year cumulative survival rate, 11.2%; 3-year cumulative survival rate, 1.9%; and 5-year cumulative survival rate, 0%) than patients without a PVTT (1-year survival rate, 88.2%; 3-year survival rate, 8.5%; and 5-year survival rate, 2.1%) (log-rank test, P<0.001).

## Discussion

To investigate the impact of clinical factors on the OS rate of patients with unresectable non-viral HCC who underwent TACE, the factors that exhibited a potential impact on the prognosis of non-viral HCC were compared. It was found that pre-existing DM, an elevated baseline NLR and the presence of a PVTT independently predicted a poor OS rate in non-viral HCC patients. In addition, the combination of DM and NLR appeared to be a stronger predictor for OS than DM or NLR alone. It was observed that DM and NLR were significantly correlated with the indicators of intrahepatic metastasis.

Several studies have confirmed that DM significantly promotes the progression of non-viral HCC (11,21). Therefore, in recent years, with the hypothesis in mind that DM may be able to predict the survival of HCC, several early studies have shown that DM is an independent factor for poorer survival in patients undergoing surgical and non-surgical treatment (17,18,22). Similar to the aforementioned studies, the present study also observed that among non-viral HCC patients who had undergone TACE, the patients with DM exhibited a poorer OS rate than those without DM. However, another study reported that the survival difference between patients with and without diabetes was not significant in a Child-Pugh B group undergoing non-surgical treatment (18). It was assumed that the inconsistent results may be caused by the difference in HCC etiology (major viral causes vs. non-viral causes) and non-surgical treatment strategy (TACE, percutaneous injection therapy vs. TACE) between the two studies. In addition, a previous study also showed that there was no significant difference in the five-year OS rate between non-viral HCC patients with DM and without DM (23). A probable reason for this divergency may be that more patients without DM were enrolled (75.4 vs. 41.7%) and due to the fact that varying treatment strategies were adopted (TACE vs. resection) in the present study. Therefore, further studies are necessary to investigate the exact role of these divergencies in the prognosis of non-viral HCC.

Although a previous study identified that the mechanism underlying effect of DM on the prognosis of HCC could be diabetes-related liver function failure (18), another study showed that DM-related insulin resistance may be involved in the hepatocarcinogenesis of non-viral HCC (24). DM has been further confirmed to positively correlate with macrovascular invasion among transplanted HCC patients (25). PVTT, which is a type of advanced macrovascular invasion, also indicates a poorer prognosis for patients with HCC (26). Elevated GGT has also been found to be a significant risk factor for microvascular invasion in patients with multinodular HCC (27). In the present study, it was found that the patients with DM exhibited a significantly higher ratio for the presence of PVTT and elevated GGT, and that DM correlated positively with them. It was inferred that DM may affect the prognosis of non-viral HCC by inducing macrovascular and microvascular invasion, particularly by forming a tumor thrombus in the portal vein.

In patients with cancer, systemic inflammation measured by NLR plays crucial roles at various stages of HCC tumorigenesis (14,15). A few studies have demonstrated that the NLR could predict the OS rate in HCC patients undergoing surgical and non-surgical treatment (19,28). An elevated NLR can signify that relative neutrophilia is correlated with more aggressive tumor behavior in uterine cancer (29). Neutrophils also promote tumor invasion by secreting neutrophil-derived hepatocyte growth factor and by promoting the adhesion and motility of cancer cells (30–32). As in the aforementioned studies, the present study confirmed that an elevated baseline NLR predicted a poorer OS rate in patients with non-viral HCC. Additionally, an elevated NLR was correlated with tumor multiplicity and the presence of a PVTT, which were indicators of the intrahepatic metastasis of HCC. These data indicate that an elevated baseline NLR may result in a poorer outcome of HCC through neutrophil-promoted intrahepatic metastasis.

The carcinogenic effects of insulin resistance in HCC may be the activation of insulin-like growth factor (IGF)-1 and 2 stimulated by compensatory hyperinsulinemia (33). IGF-1 may stimulate cancer angiogenesis in part by upregulating vascular endothelial growth factor (VEGF) expression (34), whereas IGFs (IGF-1 and IGF-2) promote vasculogenesis in embryonic stem cells through the upregulation of VEGF (35). All these studies indicate that VEGF is extremely significant in the carcinogenesis of HCC. Besides these findings, other data has indicated that VEGF released by neutrophils also promotes angiogenesis in the progression of cancer, including HCC (36). In the present study, it was observed that a combination of DM and NLR could predict a stepwise alteration in the OS rate of non-viral HCC. Since DM and neutrophils are closely associated with VEGF, we hypothesize that pre-existing DM together with an elevated NLR may affect the survival rate of non-viral HCC through the VEGF angiogenesis pathway, and a combined use of these two factors may be a more valuable prognostic indicator for non-viral HCC patients undergoing TACE.

The present study confirmed that the presence of a PVTT predicted a poor survival rate in patients with HCC, as published previously (26). While the previous study (26) demonstrated the predictive power of the presence of a PVTT in virus-related HCC, the present study also observed that the presence of a PVTT exhibited a prognostic power on non-viral HCC. Although GGT level, tumor multiplicity and tumor diameter have been identified as independent prognostic factors for HCC (37,38), the multivariate analysis in the present study could not identify a GGT level of >50 U/l, multiple tumors or a tumor diameter of ≥5 cm as independent factors. The reason for this may be that pre-existing DM, an elevated baseline NLR and the presence of a PVTT exhibited a stronger prognostic impact than GGT, tumor multiplicity or tumor diameter on the progression of non-viral HCC.

There are certain limitations to the present study. First, the study was a retrospective observational study with a lack of Child-Pugh score follow-up, which is an indicator of hepatic functional reserve. Therefore, although several studies have shown that NLR and DM affected the long-term survival of HCC partly through their effect on hepatic functional reserve (18,38), the effect of DM and NLR on the survival of non-viral HCC through their impact on hepatic reserve cannot be confirmed. Furthermore, due to the lack of recurrence of a follow-up, the association between DM, NLR and liver cancer specific survival cannot be verified. Finally, the present study was confined to a single institution, and it is possible that the associations observed were due to chance. Confirmatory prospective studies in larger cohorts, including multiple institutions, are required.

In conclusion, despite these limitations, the present study demonstrated that pre-existing DM and an elevated baseline NLR are independent prognostic indicators of the OS rate in non-viral HCC treated with TACE. The combination of DM and NLR appears to be a stronger predictor for the OS rate than DM or NLR alone. Pre-existing DM and an elevated baseline NLR may affect the OS rate by promoting the malignant progression of HCC. Further clinical studies are required to clarify a more specific biological mechanism underpinning the role of DM and NLR in non-viral hepatocarcinogenesis.

## Figures and Tables

**Table I:** Baseline characteristics of all patients.

Characteristics	Value
Age (years)	56.8±12.5
Gender (M/F)	99/39
Presence of DM (yes/no)	34/104
NLR (≥5/<5)	46/92
Child-Pugh class (A/B)	105/33
Maximum tumor diameter (cm)	8.7±4.5
No. of tumors (single/multiple)	63/75
Presence of PVTT (yes/no)	67/71
No. of TACE procedures (1/≥1)	115/23
ALT (U/l)	51.7±67.1
AST (U/l)	70.6±85.2
Albumin (g/l)	38.9±5.3
TBIL (μmol/l)	17.9±24.8
ALP (U/l)	200.4±170.3
GGT (U/l)	201.0±230.0
AFP (ng/ml)	7697.3±21041.3
Platelet count (x10^9^/l)	189.9±96.1
INR	1.06±0.11
NLR	4.55±2.71

Values are presented as mean ± standard deviation or n. DM, diabetes mellitus; M, male; F, female; NLR, neutrophil to lymphocyte ratio; PVTT, portal vein tumor thrombus; TACE, transarterial chemoembolization; ALT, alanine aminotransferase; AST, aspartate aminotransferase; TBIL, total bilirubin; ALP, alkaline phosphatase; GGT, γ-glutamyl transpeptidase; AFP, α-fetoprotein; INR, international normalised ratio.

**Table II:** Univariate and multivariate analysis of prognostic factors for OS rate by Cox’s regression model.

	Univariate analysis		Multivariate analysis	
		
Variable	HR (95% CI)	P-value	HR (95% CI)	P-value
GGT (U/l)				
Normal (n=36)	1.519 (1.019–2.265)	0.040	1.085 (0.707–1.666)	0.707
Elevated (n=102)				
Maximum tumor diameter				
<5 cm (n=30)	1.723 (1.114–2.665)	0.014	1.379 (0.873–2.178)	0.168
≥5 cm (n=108)				
No. of tumors				
Single (n=63)	1.483 (1.038–2.119)	0.030	1.119 (0.759–1.650)	0.570
Multiple (n=75)				
Presence of PVTT				
Absence (n=71)	4.336 (2.955–6.363)	<0.001	4.235 (2.787–6.436)	<0.001
Presence (n=67)				
Presence of DM				
Absence (n=104)	1.868 (1.239–2.816)	0.003	1.843 (1.190–2.854)	0.006
Presence (n=34)				
NLR				
<5 (n=92)	2.136 (1.466–3.114)	<0.001	2.126 (1.429–3.165)	<0.001
≥5 (n=46)				

GGT, γ-glutamyl transpeptidase; PVTT, portal vein tumor thrombus; DM, diabetes mellitus; NLR, neutrophil to lymphocyte ratio; HR, hazard ratio; CI, confidence interval; OS, overall survival.

**Table III:** Significant Spearman’s correlation coefficients (ρ) for patients with or without DM and for those with baseline/elevated NLR and the presence of PVTT or elevated GGT.

	With DM/without DM			NLR≥5/NLR<5	
			
Variable	ρ	P-value	Variable	ρ	P-value
Presence of PVTT	0.218	0.01	Multiple tumors	0.185	0.03
Elevated GGT	0.186	0.03	Presence of PVTT	0.174	0.04

PVTT, portal vein tumor thrombus; GGT, γ-glutamyl transpeptidase; DM, diabetes mellitus; NLR, neutrophil to lymphocyte ratio; GGT, γ-glutamyl transpeptidase.

## Abbreviations

DM: diabetes mellitus
HCC: hepatocellular carcinoma
OS: overall survival
TACE: transarterial chemoembolization
HBV: hepatitis B virus
HCV: hepatitis C virus
NLR: neutrophil to lymphocyte ratio
PVTT: portal vein tumor thrombus
AFP: α-fetoprotein
GGT: γ-glutamyl transpeptidase
INR: international normalized ratio
VEGF: vascular endothelial growth factor
